# Embryonic stages in cerebellar afferent development

**DOI:** 10.1186/s40673-015-0026-y

**Published:** 2015-06-11

**Authors:** Maryam Rahimi-Balaei, Pegah Afsharinezhad, Karen Bailey, Matthew Buchok, Behzad Yeganeh, Hassan Marzban

**Affiliations:** Department of Human Anatomy and Cell Science, College of Medicine, Faculty of Health Sciences, University of Manitoba, Rm129, BMSB, 745 Bannatyne Avenue, Winnipeg, Manitoba R3E 0J9 Canada; College of Medicine, Faculty of Health Sciences, Manitoba Institute of Child Health (MICH), University of Manitoba, Winnipeg, Manitoba Canada; Program in Physiology and Experimental Medicine, Hospital for Sick Children and University of Toronto, Toronto, Ontario Canada

**Keywords:** Mossy fiber, Climbing fiber, Neuromodulatory fiber, Cerebellar primordium

## Abstract

The cerebellum is important for motor control, cognition, and language processing. Afferent and efferent fibers are major components of cerebellar circuitry and impairment of these circuits causes severe cerebellar malfunction, such as ataxia. The cerebellum receives information from two major afferent types – climbing fibers and mossy fibers. In addition, a third set of afferents project to the cerebellum as neuromodulatory fibers. The spatiotemporal pattern of early cerebellar afferents that enter the developing embryonic cerebellum is not fully understood. In this review, we will discuss the cerebellar architecture and connectivity specifically related to afferents during development in different species. We will also consider the order of afferent fiber arrival into the developing cerebellum to establish neural connectivity.

## Introduction

The cerebellum is responsible for coordinating movement and maintaining equilibrium, and it is also involved in behavioral, cognitive and emotional functions [1–4]. Proper cerebellar function depends on well-organized neuronal connections and the integration of afferent and efferent fibers throughout cerebellar circuitry [5]. The cerebellum can be divided into two longitudinal regions along the rostral to caudal axia, which are anatomically named the vermis and hemisphere. Mediolaterally, each of these regions is folded into lobes, lobules and folia. Traditionally, the cerebellum is divided into 3 lobes that are further subdivided into 10 lobules. Lobules I-X numbering is used for the lobules of the vermis and the corresponding lobules of the hemisphere are indicated with the prefix H (e.g. [6–8]). Recent studies suggest that the fundamental architecture of the cerebellum is organized into five transverse zones based on gene expression and afferent fiber termination; the anterior zone (AZ: lobules I-V in mice), the central zone (CZ: lobules VI-VII, which is further subdivided into anterior (CZa) and posterior (CZp) components [9, 10]), the posterior zone (PZ: lobules VIII-dorsal lobule IX), and the nodular zone (NZ: ventral lobule IX and lobule X) (e.g. [11–18]). The boundaries of these zones do not align with the lobes and lobules, but do correspond to the pattern of afferent termination, and thus provides a more functionally relevant way of dividing the cerebellum.

The three-layered cerebellar cortex contains six main neuronal cell types: stellate and basket cells in the molecular layer, Purkinje cell somata in the Purkinje cell layer, and granule cells, Golgi cells, and unipolar brush cells in the granular layer. Neurons of the cerebellar nuclei are located close to the roof of the fourth ventricle deep within the cerebellar white matter. The cerebellar nuclei and the lateral vestibular nucleus constitute the sole output (efferent) of the cerebellum, and play a central role in cerebellar circuitry and function (e.g. [1, 19–22]).

The cerebellum is connected to the brainstem by three pairs of peduncles (superior, middle, and inferior), which are the pathways by which cerebellar afferents and efferents enter and exit [23, 24]. Cerebellar afferents can be grouped into two major types: mossy fibers and climbing fibers. Mossy fibers constitute the majority of afferent fibers in the adult cerebellum and arise from multiple sources. There are four main groups of mossy fibers that project to specific parts of the cerebellum: 1) the somatosensory projections (spinocerebellar and trigeminocerebellar fibers), which originate from the spinal cord and the trigeminal system (ganglion and nuclei) respectively, and project primarily to lobules I-VI and lobule VIII (e.g. [23, 25–27]); 2) vestibulocerebellar projections, which originate from the vestibular system (ganglion and nuclei) and terminate in ventral lobules IX and the flocculus with the adjacent ventral flocculonodular [28, 29]; 3) reticulocerebellar projections, which originate from the lateral reticular nuclei, the paramedian reticular nucleus and the nucleus of Roller, and nucleus reticularis tegmenti pontis terminate throughout the cerebellum, but generally mirror the projections of the spino/trigeminocerebellar and vestibulocerebellar systems (e.g. [30–33]) pontocerebellar projections, which originate from the pontine nuclei and terminate throughout the cerebellum, especially in the vermis and hemispheres of lobules VI-VII (central zone), but not in the nodulus and flocculus [34–36]. The nucleus prepositus hypoglosi is also a source of a mossy fiber system that projects restricted regions of crura I and II as well as the caudal vermis and vestibulocerebellum [37]. Mossy fiber afferents communicate with cerebellar nuclei neurons and with Purkinje cells through granule cells/parallel fibers [24, 38–40]. Climbing fibers are exclusively derived from the inferior olivary complex and enter the contralateral cerebellum through the inferior cerebellar peduncle [41, 42]. Climbing fibers directly synapse with the cerebellar nuclei and Purkinje cells (the sole output cells of the cerebellar cortex) [43–45]. In addition, a third set of afferents not categorized as mossy or climbing fibers exists, which terminate in all three layers of the cerebellar cortex and the cerebellar nuclei. Their functional role is neuromodulation and thus, they are categorized as neuromodulatory cerebellar afferents (i.e., serotonergic, noradrenergic, acetylcholinergic, dopaminergic, and histaminergic neuromodulatory afferents) (e.g. [1, 46–49]).

Extension and direction of early afferent fibers (axon pioneers) to their target neurons during development is controlled by genes and molecules, which mostly play a role in repulsion attraction (e.g. [50]). Several families of these proteins have been identified and among them four well-characterized families play an important role in axon guidance and development such as; Ephrin/Eph [50, 51], Semaphorins/ Neuropilins, [52, 53], Netrin [54, 55], and Robo/Slit [56, 57]. Molecules, such as integrins, fasciclin and neural cell adhesion molecules (NCAMs), are important in pioneer axon development by providing a substrate that promotes outgrowth of growth cones [58]. In addition, morphogens, initially characterized based on their effects on early patterning, are being increasingly implicated in axon guidance [50]. Morphogens found to be essential in cerebellar development include the Wnts family [59], Sonic hedgehog (Shh) [60, 61] and bone morphogenetic proteins (BMPs) [62]. These molecules from cell surfaces and intracellular signaling pathways provide cues that enable growing axons to terminate at their target cells. The Eph/Ephrin gene family has been indicated as a possible molecular pathway involved in regulating afferent patterning in both chick and mouse embryonic cerebella [63–65]. Engrailed-2 is important in development of cerebellum and regulates mossy fiber projections [66, 67]. UNC-5, a Caenorhabditis elegans protein that is essential for dorsal guidance of pioneer axons is important in development of the rostral cerebellum [68]. It has also been suggested that axonal development in the cerebellum is regulated by WNT-7a signaling [69]. Furthermore, gene mapping studies have shown that precerebellar nuclei including inferior olivary nuclei neurons likely originate from an area expressing Ptf1a, Oli3 and Wnt1 (e.g. [70]). Brn3 expression has been shown to be necessary for inferior olivary nuclei development [71]. Gene expression appears to be conserved across vertebrates as the inferior olivary nuclei of zebrafish also express ptf1a and brn3a [72]. It is known that the cerebellum and inferior olive express neurotrophins and their receptors that are involved in climbing fiber development [73]. It is also known that some genes are expressed differently in subsets of mossy fibers (e.g. somatostatin) and climbing fibers (e.g. corticotropin releasing factor) [74]. However, the regulatory roles of these genes and proteins in cerebellar afferent projections are poorly understood and will be interesting to investigate further to understand cerebellar circuit formation during cerebellar development.

The basic organization of afferent and efferent cerebellar connections appears to be conserved throughout evolution [75]. While there is much information regarding sources, pathways, terminations, and functions of afferents in the adult cerebellum, little attention has been paid to the subject during embryonic development. The order of afferent fiber arrival during cerebellar development is likely to be important in setting up the principal cerebellar circuits, but this is poorly understood. The basic spatiotemporal pattern of cerebellar afferents appears to be common between mammals, avians, reptiles, amphibians, and fish (e.g. [76, 77]). Therefore, in this review, we integrate information from different species in an attempt to resolve the order of afferent fiber arrival into the developing cerebellum.

## Review

### Development of cerebellar afferents

#### Mossy fibers

Mossy fiber projections probably play a critical role in establishing and organizing cerebellar circuitry during early embryonic development. Conventionally, it is believed that mossy fibers enter the early embryonic cerebellum and target the Purkinje cells. However, it has been demonstrated that mossy fiber contacts with Purkinje cells are temporary and transient. Postnatally, as the granular layer matures, mossy fibers displace from Purkinje cells and synapse with their adult targets, the granule cell dendrites (e.g., [14, 77–81]). This multi-stage process of mossy fiber relay on timing and order of arrival during cerebellar development could be required for proper circuit formation and/or for afferents to pattern the target tissue.

### Somatosensory pathway

Somatosensory input is carried into the cerebellum via spinocerebellar and trigeminocerebellar tracts.

#### Spinocerebellar afferents

The spinocerebellar afferent system is comprised of the dorsal spinocerebellar, ventral spinocerebellar, cuneocerebellar, rostral spinocerebellar tracts and from the central cervical nucleus which collectively convey proprioceptive information to the cerebellum. However, dorsal spinocerebellar tract component from lamina V and cuneocerebellar component from the internal cuneate nucleus transmits exteroceptive information to the cerebellum. The spinocerebellar afferents originate from diverse laminae of the spinal cord and can be classified into crossed and uncrossed tracts according to the cell origin and fiber course [82]. The cerebellum receives proprioceptive information from the hind limbs through the dorsal spinocerebellar tract (uncrossed) and the ventral spinocerebellar tract (crossed) [82, 83]. The dorsal spinocerebellar tract originates from the dorsal horn (mainly dorsal thoracic nucleus that is also known as Clarke’s column) and provides a major mossy fiber input to the spinocerebellum, which plays a significant role in the control of posture and locomotion [84, 85]. The input conveyed by the ventral spinocerebellar tract originates from neurons of the dorsal and ventral horn and it monitors activity of spinal interneuronal networks [82, 86, 87]. The cuneocerebellar and rostral spinocerebellar tracts are the upper limb equivalents of the dorsal and ventral spinocerebellar tracts, respectively. The sources of these tracts are well known: the cuneocerebellar tract originates from the external and internal cuneate nucleus and the gracile nucleus in the medulla oblongata and the rostral spinocerebellar tract originates from the cervical spinal cord. The termination of all spino- and cuneocerebellar tracts is in bilateral, alternating symmetrically disposed longitudinal aggregates of mossy fiber terminals. The spinocerebellar afferents project to the cerebellum through the inferior cerebellar peduncle (non-crossed) and the superior cerebellar peduncle (crossed), and terminate in the anterior zone (lobules I-V) and posterior zone (lobules VIII-IXd) [28, 88].

Clinically, the spinocerebellar system is involved in a heterogeneous group of disorders classified collectively as spinocerebellar ataxia [89]. Spinocerebellar ataxia is a neurodevelopmental and neurodegenerative disease characterized by progressive incoordination of movement and degeneration of the cerebellar cortex and spinocerebellar pathways [89, 90]. It is believed that the cerebellar circuit organization, which is established during early cerebellar development, may be altered in ataxia (e.g. [91]).

#### Development of the spinocerebellar afferents

Spinocerebellar mossy fiber projections to the cerebellum during prenatal development have been studied in mice using *in vitro* tract tracing techniques (e.g. [22, 92]). Results of these studies have shown that at embryonic day 12/13 (E12/13), neuronal sources of spinocerebellar mossy fibers from the caudal cervical spinal cord are present, but there are no projections to the cerebellum. At approximately E13/14, spinocerebellar projected fibers have been shown to be present in the rostrolateral portions of the cerebellum adjacent to the isthmus region. Spinocerebellar fibers progress farther into the cerebellum and the number of crossed fibers increases as aging occurs [22]. In rats, tract-tracing methods have shown that spinocerebellar fibers project to the cerebellum in two successive groups: the first group is present in the cerebellum perinatally and the second group reaches the cerebellum by postnatal day 3 (P3) and targets the vermis of the anterior lobe and pyramis [93]. However, this timing was revised by Ashwell and Zhang, who showed that spinocerebellar fibers may be present in the developing rat cerebellum at around E15 [92, 94]. The external cuneate neurons are generated from the caudal rhombic lip between E13 and E15 in mice and may enter into the cerebellum during E13-E15 and thereafter [7, 95]. In the rat, cuneocerebellar fibers arrive in the developing cerebellum by E16/17 ipsilaterally via the inferior cerebellar peduncle [6].

In the chicken, spinocerebellar projections have been studied by injecting WGA-HRP into the spinal cord of embryos at different stages of development. These studies showed that spinocerebellar fibers entered the cerebellum at around the 7th incubation day [96]. In the African clawed frog, *Xenopus laevis,* spinocerebellar afferents were studied using HRP tracer techniques. At approximately stage 50, before the formation of the limbs, the ventral spinocerebellar projections appeared to be present in the cerebellum prior to entry of the dorsal spinocerebellar tract [97]. Although primary spinocerebellar projections from spinal ganglion cells to the cerebellum have been demonstrated in the adult frog [98, 99], they were not detectable during development [97]. Tract tracing methods have also been used to study spinocerebellar projections in reptiles including turtles (*Pseudemys scripta elegans* and *Testudo hermanni*), lizards (*Varanus exanthematicus*), and snakes (*Python regius*). The basic pattern of cerebellar afferent projections appears to resemble that of other vertebrates. However, it has been suggested that there is no column of Clarke or central cervical nucleus in the reptilian spinal cord [99]. To our knowledge, no developmental studies have investigated spinocerebellar projections in reptiles. In the lesser spotted dogfish (*Scyliorhinus*), spinocerebellar afferents were shown to project to the developing cerebellum at around stage 32 (equivalent to around E14.5 in the mouse) [100, 101].

In summary, spinocerebellar fibers invade the developing cerebellum at around E13/14 in the mouse (e.g. [22, 92]), E15 in the rat [94], HH33 (Hamburger-Hamilton stages 33) in the chicken (around the 7th incubation day; [96]), stage 50 in *Xenopus laevis* [97], and at stage 32 in the dogfish [101, 102] (Tables 1 and 2).

**Table 1:** Timing of cerebellar afferent fiber arrival in different species during the embryonic period

Afferent fiber	Mouse	Rat	Chicken	Xenopus	Reptile	Fish^a^
Spinocerebellar a	E13/14	E15	HH33/E7	Stage 50	n/a	Stage 32
Trigeminocerebellar a	n/a	E22/P0	n/a	Stage 48	n/a	Stage 32
Vestibulocerebellar a	E11-13	13-14/15	n/a	Stage 48	n/a	n/a
Reticulocerebellar a	E13/14	E16/17	n/a	Stage 48	n/a	Stage 32
Pontocerebellar a	E16	perinatal	n/a	n/a	n/a	n/a
Olivocerebellar a	E14/15	E17	E9	n/a	n/a	Stage 32
Serotonergic cerebellar a	postnatal	postnatal	n/a	Stage 56	n/a	n/a
Noadrenergic cerebellar a	E14	E17	n/a	n/a	n/a	n/a
Cholinergic cerebellar a	n/a	n/a	n/a	n/a	n/a	n/a
Dopaminergic cerebellar a	n/a	n/a	n/a	n/a	n/a	n/a
Histaminergic cerebellar a	n/a	n/a	n/a	n/a	n/a	n/a

Note: a, afferent fiber

The “n/a” means that there is no data available for afferents projection to the cerebellum during embryonic development in these species

^a^This is the only stage that has been studied; therefore everything arrives at stage 32

**Table 2:** Timing of cerebellar afferent fiber arrival in different species during embryonic and postnatal stages

Afferent fiber	Embryonic									postnatal	
Days	2	4	6	8	10	12	14	16	18	0/2	adult
Spinocerebellar a							►				
Trigeminocerebellar a											
Vestibulocerebellar a						►					
Reticulocerebellar a							►				
Pontocerebellar a								►			
Olivocerebellar a							►				
Serotonergic cerebellar a										►	
Noradrenergic cerebellar a							►				
Cholinergic cerebellar a											
Dopaminergic cerebellar a											
Histaminergic cerebellar a											

a, afferent fiber

#### Trigeminocerebellar afferents

The trigeminal system (trigeminal nerves, ganglion, and nuclei) is the largest cranial nerve, which has both sensory and motor components [103]. Peripherally, the trigeminal nerve is comprised of three branches: ophthalmic (V1, sensory), maxillary (V2, sensory), and mandibular (V3, sensory and motor), which converge together on the trigeminal ganglion. From the trigeminal ganglion, a single large sensory root enters the brain stem at the level of the pons and terminates at the complex trigeminal nuclei, which consist of the spinal trigeminal nucleus, main (principal) trigeminal nucleus, and mesencephalic trigeminal nucleus. The motor component is derived from the basal plate of the embryonic pons, while the sensory component originates from the alar plate and the neural crest (e.g. [104]).

The trigeminal nerve carries temperature, pain and tactile information from the skin, jaws and teeth. In addition, it carries proprioceptive input primarily from the muscles of mastication to the mesencephalic trigeminal nucleus, which projects to higher centers to coordinate jaw movement in activities such as suckling, mastication, biting and speech (e.g. [105]). In adult rats, the mesencephalic and interpolaris trigeminal nuclei have direct projections to the cerebellum [106, 107]. There are also primary trigeminal afferent projections from the ganglion (probably from the mandibular branch) to the cerebellum of adult rats [108]. The primary trigeminocerebellar projections (originating from the trigeminal ganglion) run through the superior peduncle, whereas secondary trigeminocerebellar projections (originating from the trigeminal nuclei) run through the inferior peduncle, and both terminate in the same target areas in the cerebellum [26, 109].

In the cerebellum, the mesencephalic tract nucleus projects to the anterior lobe, the simple lobule (HVI), lobules VI, VIII, and the dorsal paraflocculus (e.g. mice [27], rats [109], and sheep [110]). The ventral group of the main (principal) and spinal tract nuclei project to all lobules in the vermis and hemispheres, whereas the dorsal parts of these nuclei have a more restricted projection field including the vermis of the lobules VI, VII, and IX and corresponding hemispheres [110]. In the rat, the trigeminal nucleus oralis projects to ipsilateral orofacial portions of four major tactile areas (crura I and II, the paramedian lobule, and the uvula) of the cerebellar cortex [111, 112]. Retrograde tracing has shown that most of the spinal trigeminal nuclei project to the paramedian lobule in the tree shrew (*Tupaia glis*), but the principal and mesencephalic trigeminal nuclei do not [113]. Trigeminocerebellar afferents have been demonstrated in *Xenopus laevis* [114]. In mallards, the trigeminocerebellar afferents terminate predominantly in lobules V, VI and VII, and possibly in lobule IV, and are ipsilateral except for the vermal area [115]. The secondary trigeminocerebellar projections have been demonstrated in mammals (e.g. [116]), birds [117], reptiles [100], and amphibians [114].

#### Development of the trigeminocerebellar afferents

In the central nervous system, mesencephalic neural crest cells emerge and start migrating at the 4- to 7-somite stage in mice, some of which give rise to the primary sensory neurons that form the mesencephalic trigeminal nucleus [104]. At E8.5, by the 10-somite stage, the first group of neurons can be identified in the rostral part of the mesencephalon [104]. This is the earliest neuronal development that has been detected in the central nervous system; this is in contrast to previous information, which indicated that neurons first differentiated in the mouse central nervous system by E9-E10 [104]. It has been demonstrated that the cells of the mesencephalic trigeminal nucleus are born before E11 in rats [118], and as early as E8 in mice [104]. Although formation of this nucleus takes place during early neural tube development, Ashwell and Zhang have suggested that the trigeminocerebellar afferents project to the cerebellum around E22 in rat [94].

Information regarding the development of trigeminal projections in the avian cerebellum is limited. In reptiles, the presence of trigeminocerebellar projections has been demonstrated [100], but not studied during development. In amphibians (*Xenopus laevis*), the secondary trigeminocerebellar projection (from the descending nucleus of the trigeminal nerve) has been shown in the cerebellum at approximately stage 48 [97]. In addition, trigeminocerebellar fibers can be observed in the cerebellum at stage 32 in the dogfish [102] (Tables 1 and 2). Thus, the trigeminal system components seem to be among the earliest neurons and fibers to develop in the vicinity of the cerebellar primordium. However, the lack of clarity in the timing of the developing trigeminocerebellar projections requires further research.

### Vestibulocerebellar afferents

The vestibulocerebellar system, which is important for maintaining balance and equilibrium, provides sensory input to the cerebellum [119]. The vestibular nucleus includes four subnuclei: superior, inferior, medial, and lateral [119]. The lateral vestibular nucleus does not receive vestibular root fibers, but Purkinje cell axons from the B zone (the lateral part of the vermis), therefore, it is a cerebellar rather than a vestibular nucleus [28]. Projections from the vestibular system to the cerebellum are comprised of two components: primary vestibular mossy fiber afferents (i.e. originate from the vestibular ganglion) and secondary vestibular mossy fiber afferents (i.e. originate from the vestibular nuclei) [120]. Vestibular nerve projections to the cerebellum are primarily limited to the nodulus, uvula, and flocculus [120, 121]. Additionally, in bottom deep fissures (basal part of the lobules), lobules I and II, where they are distributed in parallel longitudinal aggregates [122], the flocculus does not receive primary vestibular mossy fibers [123]. The “vestibular commissure” of Larsell would form the basis of the flocculonodular lobe [124]. Primary and secondary vestibulocerebellar afferents have been shown in amphibians [125, 126], reptiles [100, 127], birds [128] and mammals (e.g. [116, 129–131]).

#### Development of vestibulocerebellar afferents

It is suggested that vestibular ganglion neurons are born on E10-E14 in mice [131], and E11-E13 in rats [132]. Vestibular nuclei projection neurons are born in the neuroepithelium lining the fourth ventricle between E12 and E14 in the rat in the following order: the lateral (approximately E12), superior (approximately E13), inferior (approximately E13-14), and medial vestibular nucleus (approximately E13-14) [132, 133]. Historically, the classical observation of Tello and Ramon Y Cajal (1909) suggested that vestibular fibers were the first afferents to reach the cerebellum at six days prenatal in the mouse. However, the timing was slightly revised some years later, with vestibular axons from the vestibular ganglia being the first afferents to enter the cerebellum at E13-14/15 in the rat and E11-12/13 in mice [94, 133, 134]. From E15, collateral branches from primary vestibulocerebellar afferents form contacts with the developing vestibular nuclei within the subventricular zone [134].

Vestibular nuclei are generated at around E2 in chickens [135]. However, to date there are no data for avian vestibulocerebellar fiber development. Developmental studies are currently lacking on reptilian vestibulocerebellar projections, but in amphibians (*Xenopus laevis*), it has been demonstrated that vestibulocerebellar projections, which mainly arise bilaterally in the nucleus vestibularis caudalis, appear at around stage 48 [97] (Tables 1 and 2). Although the vestibulocerebellar projections are suggested to be the earliest afferents to invade the cerebellar primordium, further studies using a greater range of experimental approaches are required to confirm this important and fundamental process.

### Reticulocerebellar afferents

A mixture of neurons and nerve fibers located in the brainstem make up a poorly defined group of nuclei collectively referred to as the reticular formation. The reticular formation is a primitive neuronal network upon which a more morphologically organized mass of neurons have developed during evolution [135, 136]. The reticular formation can be divided into three bilateral longitudinal columns based on both structure and function: median (raphe nuclei), medial, and lateral nuclei [136]. The lateral reticular nucleus can be further divided into three subnuclei (parvocellular, magnocellular and subtrigeminal) that project to the cerebellum. The caudal and ventral part of the lateral reticular nucleus projects bilaterally, mainly to the vermis, its rostral and dorsal part, that receives the “ipsilateral forelimb tract” and a collateral projection of the rubrospinal tract projects to the ipsilateral hemisphere [137]. The paramedian pontine reticular nucleus projects bilaterally to the cerebellar lobules VI, VII and the crura I and II that symmetry about the medline [32]. The parvocellular portion and adjacent magnocellular portion project primarily to the vermis, the remaining magnocellular subnucleus projects to the hemispheres, and the subtrigeminal subnucleus projects to the flocculonodular lobe. Neurons of the reticular formation project to the cerebellar cortex and cerebellar nuclei through the superior/middle/inferior cerebellar peduncles [138]. Reticular formation projections, which are aligned with the cerebellar architecture, carry out functions such as orofacial behaviour, including eye blink reflexes [39, 138].

#### Development of reticulocerebellar afferents

In the rat, the reticulocerebellar nucleus is not identifiable until birth when the axons of these neurons reach the cerebellum, even though reticular nucleus neurons are born on E12/13 [30]. Other studies have suggested that axons from the lateral reticular nuclei begin arriving in the developing cerebellum by E16/17 in rat (equivalent to E13/14 in mouse; [7]) via the inferior cerebellar peduncle [139]. At present, detail regarding the reticulocerebellar projections in the avian cerebellum is lacking (e.g. [96]). In reptiles, projections to the cerebellum were demonstrated from the reticular formation [100], but developmental studies in this system are currently required. Reticulocerebellar connections have been demonstrated experimentally at stage 48 in *Xenopus laevis*, [97]. In the dogfish, reticulocerebellar neurons are observed at stage 32 from the reticular formation of the mesencephalon and rhombencephalon [101, 102] (Tables 1 and 2).

The reticular formation is a complicated organization of nuclei that is involved in both sensory and motor pathways, and is generally one of the earliest nuclei to grow ontogenetically and phylogenetically. It seems that the reticulocerebellar projections are established early in development as a fundamental requirement to establish the basic functional circuitry of this pathway.

### Pontocerebellar afferents

The pontine nuclei, located at the basilar portions of the pons (basilar pontine nuclei), receive most of their input from the cerebral cortex [140]. The majority of mossy fibres innervating the hemispheres originate from the pontine nuclei [138]. All pontocerebellar fibers end as mossy fibers in the cerebellar nuclei and cortex [141, 142]. The nucleus reticularis tegmenti pontis project to the cerebellar nuclei (pronounced in lateral and posterior interposed nuclei), the paraflocculus and crus II of the ansiform lobule [143]. The size of the pontine nuclei increases in parallel with the size of the associated cerebral cortex, reaching peak development in humans [136].

#### Development of pontocerebellar afferents

Pontine neurons arise from the lower rhombic lip around E14 to E17 and tangentially migrate to the ventral pons in the rat. At E16.5, migrating pontine neurons are present in the pontine nuclei with their axons laterally projecting towards the developing cerebellum [95]. These axons cross the midline and enter the cerebellum through the middle peduncle [144]. The neurons of the pontine nucleus are generated from the rhombic lip at E16 in mice [145], while in the rat, pontocerebellar nuclei are first labelled at birth [92]. It has been suggested that pontine mossy fibers enter the cerebellum perinatally in rat [92, 146]. The pontocerebellar projections are found in birds and mammals [33, 128, 145–148], but projections from the primordial pontine nuclei to the cerebellum in reptiles and amphibians have not been demonstrated [100] (Tables 1 and 2).

### Climbing fibers

Climbing fibers are the second major type of cerebellar afferents. Unlike mossy fibers, which originate from numerous sites in the nervous system, climbing fibers originate exclusively from the inferior olivary nucleus (e.g. [41, 149–151]). The inferior olivary nucleus can be subdivided into three major subnuclei: the principal olivary nucleus, which projects mainly to the cerebellar hemisphere, the dorsal accessory olivary nucleus, which projects to the vermis and hemispheres, and the medial accessory olivary nucleus, which projects to the vermis and paravermis in the rat [152]. Climbing fibers relay information to the cerebellum from several regions, such as the cerebral cortex, thalamus, red nucleus, reticular formation, trigeminal nuclei, and spinal cord. It is evident that inferior olivary neurons are necessary for proper cerebellar function, possibly in motor learning [153] and in motor timing [154]. The inferior olivary nucleus is a common feature of amphibians, reptiles, birds and mammals. Inferior olivary nucleus projections have been demonstrated experimentally in the frog [155, 156], and termination of these afferents in a longitudinal pattern has been demonstrated in the avian cerebellum [157]. The olivocerebellar circuit is essential for normal cerebellar function [158].

#### Development of olivocerebellar afferents

Several studies support that inferior olivary neurons originate from the caudal region of the rhombic lip, r6-8 (reviewed in [70]). Gene mapping studies have shown that IO neurons likely originate from an area expressing Ptf1a, Oli3 and low levels of Wnt1 (reviewed in [70, 159]. Brn3 expression has been shown to be necessary for ION development [71]. Post-mitotic inferior olivary cells in the caudal hindbrain migrate circumferentially toward the ventral floor plate of the medulla where the mature inferior olivary nuclei will form [139, 160]. Inferior olivary neurons originate at E10-11 in mice [70], E12-14 in rats [160], and E3-5 in chicks [161].

Axons of inferior olivary neurons begin migration before the cell bodies, such that the axons reach the cerebellum around the same time that the cell bodies settle in the ventral floor plate [162]. Organization of the inferior olivary subnuclei is clear by E10 in chicks [159] and E18/19 in rats [42, 139]. Olivocerebellar axons cross the midline and enter the contralateral cerebellum through the inferior cerebellar peduncle [42]. These axons reach the cerebellum around E14/15 in mice [163], E17 in rats [7, 164], and E9 in chicks [165]. Within the cerebellum, inferior olivary axons give off thick and thin branches. The thick branches form the climbing fibers, which terminate in the cerebellar cortex and synapse directly with Purkinje cells [42]. Immature climbing fibers have been shown to occur in the Purkinje cell region as early as P0 in mice [9, 166], and synapses have been found to occur as early as E19 in rats [167]. Early in postnatal development, each Purkinje cell is innervated by multiple climbing fibers. In rats, this occurs as early as P3 and peaks at P5, with an average of 3.5 climbing fibers per Purkinje cell [168, 169]. Climbing fiber innervation is subsequently reduced leaving a single strong excitatory synapse [163]. Mono-innervation occurs by P15 in rats [169]. In mice, this process is longer, ending around the third postnatal week, with defined early and late stages [151, 170]. The thin branches of the inferior olivary neurons terminate on the cerebellar nuclei [171]. In the North American opossum (*Didelphis marsupialis virginiana*), olivary axons reach the developing cerebellum at approximately postnatal day 4 [172].

From an evolutionary perspective, the lamprey, an ancient jawless fish/agnathan, possesses a region comparable to the cerebellum [173]. The presence of the inferior olivary nuclei in agnathans has not been demonstrated; however, the presence of the inferior olivary nuclei has been demonstrated in jawed fishes/gnathostomes (e.g. teleosts [72] and chondrichthyans [101, 102]). The inferior olivary nuclei in fish are considered to be homologous to the medial accessory olivary nucleus of mammals [174]. In the dogfish (chondrichthyan), the olivocerebellar projections develop after spinocerebellar projections [101, 102], similar to birds and mammals. These projections are first obvious during intermediate stage 32, when the first climbing fibers reach the cortex [101, 102]. In dogfish, Purkinje cells have matured by stage 32 [175], unlike mammalian development in which climbing fibers enter the cerebellum before Purkinje cells have matured. In zebrafish (teleost), the inferior olivary nucleus can be distinguished at 4 days post-fertilization in the ventromedial region of the posterior hindbrain, and climbing fibers have been shown to innervate Purkinje cells at 5 days post fertilization [72]. The development and organization of the olivocerebellar system appears to be conserved throughout vertebrates (e.g. [76, 175]). Overall, the arrival of inferior olivary projections to the cerebellum appears to happen at roughly equivalent developmental stages in fish, birds, and rodents (Tables 1 and 2).

### Neuromodulatory afferents

In addition to mossy fibers and climbing fibers, other cerebellar afferent pathways have also been discovered. These afferents originate from subnuclei of the reticular formation and are traditionally called neuromodulatory cerebellar afferents. They project to the cerebellum through the superior, middle, and inferior peduncles and in general terminate in all three layers of the cerebellar cortex. The neuromodulatory cerebellar afferents influence cerebellar circuitry in various ways and can be categorized into 5 groups based on the chemical messengers used for communication: serotonergic, noradrenergic, acetylcholinergic, dopaminergic, and histaminergic neuromodulatory afferents (e.g. [46–49]). In addition to their largely unknown function, the distribution and development of these afferents projection to the cerebellum is poorly understood.

### Cerebellar serotonergic afferents

The serotonergic fibers are the largest modulatory afferent fibers to the cerebellum [49]. A combination of retrograde transport studies and serotonin immunohistochemistry has shown that serotonergic cerebellar fibers arise from a few of the reticular formation subnuclei: the nucleus reticularis gigantocellularis, the nucleus reticularis paragigantocelularis, and also from the pontine reticular formation in the nucleus reticularis pontis oralis [176]. In rats, serotoninergic fibers project to all parts of the cerebellar cortex. However, the distribution of fibers across the cerebellum is not uniform, with different densities projecting to the granule cell and molecular layer and to different lobules. The fibers also terminate in the Purkinje cell layer. The distribution is found to be thickest in lobules VIII-X of the vermis. A dense uniform plexus of serotonergic cerebellar fiber projections is present in the cerebellar nuclei of the cat cerebellum [49, 176, 177]. It seems that serotonergic fibers have modulatory effects on Purkinje cells and cerebellar nuclei [178, 179].

From a clinical point of view, the serotonergic abnormalities in the cerebellum may be involved in patients with developmental neuropsychiatric disorders such as autism and attention deficit/hyperactivity disorder (ADHD) [180, 181]. Serotonin may disturb behavioral responses that are associated with cerebellar timing signals, such as the eye-blink reflex [49, 182].

#### Development of cerebellar serotonergic afferents

The expression of the serotonergic phenotype begins at E12 just caudal to the mesencephalic flexure, in rostral raphe nuclei [183, 184]. It is suggested that serotonergic projections to the cerebellum develop during the postnatal period [185], which coincides with the perinatal development of the cerebellar cortex. It has been shown that serotonergic fibers are present in the chicken [186], the opossum [172], and at stage 56 in the cerebellum of Xenopus (which may have originated from inferior raphe nucleus) [187] (Tables 1 and 2).

### Cerebellar noradrenergic afferents

Noradrenergic afferents are the second largest modulatory input to the cerebellum [46] and originate from the locus coeruleus [188], a system that supplies noradrenaline (norepinephrine) throughout the central nervous system. The locus coeruleus is located in the pons, which is involved in physiological functions such as arousal, sleep/wake cycles, nociception, anxiety, stress, learning and memory [189, 190]. Noradrenergic fibers project to all parts of the cerebellar cortex and are found around Purkinje cell dendrites, glomeruli, granule cell dendrites, and cerebellar nuclei [116]. The significance of noradrenergic cerebellar projections is suggested to be correlated with learning [191].

#### Development of cerebellar noradrenergic afferents

It is suggested that the locus coeruleus neurons are among the first central nervous system neurons to arise during ontogeny. In mice, these neurons originate from dorsal r1 between E10.5 to E12.5, and their axons may reach the cerebellum by E14 [192]. They establish axonal connections to their target areas while migrating into their nuclear area in 4th ventricle at around E17 in the rat (Tables 1 and 2). Noradrenergic axons are demonstrable in the developing cerebellum for the first time at E17 and the peak of innervation is reached at P10 [193]. This hyper-innervation is transitory and declines to adult values at around P20 [193].

### Cerebellar cholinergic afferents

Cholinergic input to the cerebellum is thinly dispersed, creating a diffuse plexus of beaded fibers in the cerebellar cortex and cerebellar nuclei [194–196]. It has been proved that a subset of vestibular projections to the cerebellar cortex are cholinergic [196]. However, the reticular formation is the principal source of cholinergic cerebellar afferents [48]. In the rat cerebellum, cholinergic fibers originate in the pedunculopontine tegmental nucleus, the lateral paragigantocellular nucleus, and, to a lesser extent in various raphe nuclei [48]. Cholinergic afferents terminate in the granule cell layer and molecular layer of the cerebellum in rat, rabbit, and cat [194]. The cholinergic fibers in the cerebellar nuclei form a moderately dense network, and could, in principle, have a significant effect on neuronal activity [48].

#### Development of cerebellar cholinergic afferents

Using specific binding sites for [^3^H]quinuclidinyl benzylate, Mallol et al. suggested that cholinergic fibers are present at a low level in the cerebellum at birth [197]. However, there is limited information regarding cholinergic cerebellar afferents during development.

### Cerebellar dopaminergic afferents

There are sparse dopaminergic projections to the cerebellar cortex and nuclei, which appear to originate mostly from the ventral tegmental area [198]. It has been demonstrated that most dopamine receptor proteins are present in Purkinje cells [199]. Dopamine can influence plasticity in Purkinje cells [46].

#### Development of cerebellar dopaminergic afferents

Dopaminergic neurons are born at approximately E10-E14 in mice (E12-E16 in rats) from the ventral midbrain/mesencephalon floor plate [200]. However, there is no on the development of dopaminergic cerebellar afferent projections.

### Cerebellar histaminergic afferents

Histamine plays a role as a neurotransmitter or a neuromodulator in the cerebellum. Interestingly, there are direct fiber connections from the tuberomammillary nucleus of the hypothalamus to the cerebellum [201], suggesting that histamine is involved in signal transmission from the hypothalamus to the cerebellum [202]. It has been proposed that histaminergic fiber projections to the cerebellum are important in regulating the level of behavioral arousal, and in the control of autonomic and emotional functions [46, 199]. Histaminergic cerebellar afferents have been shown to be present in the cerebellum of the rat, guinea pig and human [203–205].

#### Development of cerebellar histaminergic afferents

The first histamine-immunoreactive neurons have been demonstrated at E13 in the border of the mesencephalon and metencephalon [206]. However, additional information is needed regarding further development of histaminergic cerebellar afferents.

## Conclusions

The order in which cerebellar afferent projections appear throughout the development of embryonic mice and rats, in comparison with the other species, has shown that there is a general pattern in the arrival of cerebellar afferents during embryonic development. This suggests that the appearance of early cerebellar afferents takes place during a short period; those from the vestibular nuclei, inferior olive, trigeminal, and reticular nuclei arrive first, followed by the connections from spinal cord. Knowing the order in which cerebellar afferents arrive will provide information about the role that afferents may play during early embryonic development of the cerebellar primordium. The earliest afferent projections to the cerebellum may be involved in development, acting as a scaffold for subsequent incoming afferents, thereby establishing cerebellar circuitry and function. Limited knowledge about the order of cerebellar afferent development during the embryonic period prevents appropriate conclusions from being made, and requires further experimentation in this area. There is no developmental information available regarding some cerebellar afferents, particularly in the neuromodulatory category. Thus, further research is required to close this knowledge gap. In addition, because less sensitive methods were used in the past we suggest that the study of cerebellar afferent fiber development should be revisited by utilizing genetically encoded tools to detect the sequence of afferent projections during developing.
